# Céphalées d’allure migraineuse révélatrice d’un accident vasculaire cérébrale sur dissection carotidienne

**DOI:** 10.11604/pamj.2017.28.165.12620

**Published:** 2017-10-20

**Authors:** Rajaonarison Lala Andriamasinavalona, Rasaholiarison Nomena Finiavana

**Affiliations:** 1 HNFC, CHU de Besançon, France; 2 CHU JRB Antananarivo, Madagascar

**Keywords:** AVC, sujet jeune, IRM, Stroke, young subject, MRI

## Abstract

Dissections of cervicocephalic arteries are the main cause of stroke among the young adults. We report the case of a hypertensive and migrainous patient aged 59-years presenting with migrainous cephalea with flashes of light in his left eye and paroxysmal regressive paresthesia of the right hemicorpus. Clinical examination showed apraxia of speech associated with phonemic paraphasia. MRI of cerebral vascular territories with diffusion weighted imaging showed perivascular hyper-intense signal of the left internal carotid artery (intra-petrous portion) and T2 hyposignal suggesting hematoma in the wall with arterial occlusion at this level. MRI with diffusion and flair weighted imaging showed several ponctiform hyperintense signals at the level of the left anterior cerebral arterial territory and junctional hyperintense signals between the anterior cerebral artery and the middle cerebral artery without microbleeds at the level of the parenchyma, suggesting embolic stroke due to carotid dissection. The patient underwent curative Vitamin K antagonist anticoagulation treatment (target INR range of 2 to 3) after heparinotherapy with language therapy. At the three-month follow-up, angio MRI of the supra aortic trunk showed left intrapetrous internal carotid revascularization. Imaging plays an important role in the diagnostic confirmation of the dissections of cervicocephalic arteries and of possible stroke as well as in the immediate management and follow-up of patients.

## Image en médecine

Les dissections des artères cervico-encéphaliques représentent la principale cause d'accident vasculaire cérébral (AVC) chez l'adulte jeune. Nous rapportons le cas d'un homme de 59 ans qui consultait pour une céphalée d'allure migraineuse avec des scintillements de l'œil gauche et une paresthésie de l'hémicorps droit paroxystique et d'évolution régressive. Chez un patient hypertendu et migraineux où l'examen clinique a permis de trouver une paraphasie phonémique avec manque de mots. L'imagerie par résonance magnétique (IRM) encéphalique en séquence vasculaire a montré en un hypersignal périvasculaire de l'artère carotide interne gauche (partie intra pétreuse) en diffusion et hyposignal en T2*, en faveur d'un hématome de la paroi avec occlusion artérielle à ce niveau. Aussi, plusieurs hypersignaux ponctiformes ont été mis en évidence en diffusion et Flair au niveau territoire artère cérébrale antérieur gauche et jonctionnel entre l'artère cérébrale antérieur et moyenne gauche sans présence de microbleeds au niveau parenchymateuse, en faveur d'un AVC d'origine embolique suite à la dissection carotidienne. Une anticoagulation à dose curative par antagoniste du vitamine K a été mise en place (INR cible entre 2 à 3) après héparinothérapie avec une rééducation orthophonique. Une angio-IRM tronc supra aortique à 3 mois a montré une reperméabilisation de La Carotide interne gauche intra pétreuse. L'imagerie est d'un intérêt majeur dans la confirmation diagnostique des dissections des artères cervico-encéphaliques et de l'éventualité d'un accident cérébral ischémique mais aussi thérapeutique tant immédiate que pour la suivi de ses patients.

**Figure 1:**
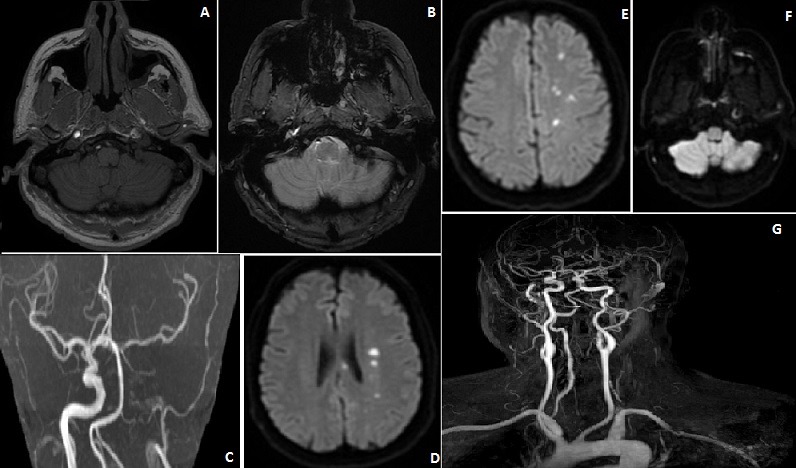
IRM encéphalique en séquence vasculaire: A) hypersignal de l’hématome pariétal de la dissection artère carotidienne gauche montré par la flèche blanche en T1; B) et en hyposignal en T2+; C) diffusion; D) occlusion des branches de la carotide interne gauche en TOF; E) reperméabilisation artère carotide interne gauche; F) et ACA gauche; G) angio IRM tronc supra aortique et artère cérébrale à 3 mois: reperméabilisation artère carotide interne interne gauche

